# Clinicopathological Significance of Exosomal Proteins CD9 and CD63 and DNA Mismatch Repair Proteins in Prostate Adenocarcinoma and Benign Hyperplasia

**DOI:** 10.3390/diagnostics12020287

**Published:** 2022-01-24

**Authors:** Kristofs Folkmanis, Elizabete Junk, Evelina Merdane, Inese Folkmane, Valdis Folkmanis, Igors Ivanovs, Janis Eglitis, Maris Jakubovskis, Sven Laabs, Sergejs Isajevs, Vilnis Lietuvietis

**Affiliations:** 1 Faculty of Medicine, University of Latvia, LV-1004 Riga, Latvia; folkmane.elizabete@gmail.com (E.J.); evelina.merdane@inbox.lv (E.M.); folkmane.inese@inbox.lv (I.F.); valdis.folkmanis@lu.lv (V.F.); igors.ivanovs@lu.lv (I.I.); janis.eglitis@lu.lv (J.E.); sergejs.isajevs@lu.lv (S.I.); 2 Department of Urology, Elbe Hospital in Stade—Teaching Hospital of Hamburg-Eppendorf University Hospital, 20246 Stade, Germany; sven.laabs@elbekliniken.de; 3 Department of Urology, East Clinical University Hospital, LV-1007 Riga, Latvia; maris.jakubovskis@aslimnica.lv (M.J.); vilnis.lietuvietis@aslimnica.lv (V.L.); 4 Department of Urology, Faculty of Medicine, Riga Stradins University, LV-1007 Riga, Latvia

**Keywords:** exosomal biomarkers, DNA mismatch repair proteins, prostate acinar adenocarcinoma, benign prostate hyperplasia

## Abstract

Introduction. Recently, it has been shown that exosomal biomarkers and DNA mismatch repair proteins (MMR) could play an important role in cancer risk stratification and prognosis assessment. The gold standard for prostate carcinoma (PCa) diagnosis is biopsy and histopathological examination. Thus, the complex evaluation of exosomal and MMR proteins could be beneficial for prostate cancer risk stratification and diagnostics. The aim of the current study was to evaluate and compare the expression of exosomal proteins CD9 and CD63 and MMR proteins in the tissue of patients with prostate benign hyperplasia (BPH) and PCa. Methods. The study was retrospective. Altogether, 92 patients with PCa and 20 patients with BPH (control group) were enrolled in the study. Exosomal and MMR protein expression was analyzed by immunohistochemistry (IHC). The follow-up for each PCa patient in our study lasted till disease progression and/or a maximum of 5 years. Results. Low-grade PCa was observed in 56 patients and high-grade PCa in 36 patients. CD63 expression was significantly higher in patients with high-grade PCa compared to those with low-grade PCa. CD9 expression was significantly downregulated in PCa patients compared to the control group. MMR protein expression deficiency was observed in 10 PCa patients. MMR proteins were maintained in all cases of BPH. The study found a negative correlation between MMR protein loss and PCa ISUP grade groups. Progression-free survival (PFS) in patients with MMR deficiency was significantly shorter than in patients with maintained MMR expression. Conclusions. CD9 protein expression was downregulated in PCa, compared to BPH, while CD63 protein expression was upregulated in high-grade PCa but downregulated in low-grade PCa. CD63 protein upregulation, CD9 downregulation, and loss of MMR protein characterized the shorter PFS of high-grade PCa patients. CD9, CD63, and MMR could be the routine immunohistochemical biomarkers for the diagnosis and risk stratification of PCa.

## 1. Introduction

Prostate cancer (PCa) remains the second-most-commonly diagnosed cancer in men [1].

Present diagnostic markers, such as prostate specific antigen (PSA), have substantial drawbacks, such as lack of disease specificity [1,2,3,4]. Tumor biopsy and histopathological evaluation is still the only definitive method of diagnosis, but it is invasive.

Different risk classification tools have been developed to distinguish patients with early PCa according to the prognosis, including the D’Amico classification system, the Cancer of the Prostate Risk Assessment score, and the National Comprehensive Cancer Network (NCCN) risk groups classification [1,2,4,5]. These systems recognize a low risk of cancer progression for PCa patients with ISUP grade group ≤ 2.

Liquid biopsies, circulating tumor cells, exosomes, and circulating nucleic acids have been developed as minimally invasive assays to monitor PCa patients [1,6,7].

Recently, there has been remarkable progress in understanding the role of exosomes in cancer development and progression. Previous studies have shown that exosomes from cancer cells might be associated with intracellular communications involved in the development of the tumor microenvironment, such as metastatic environment formation and angiogenesis, resulting in the progression of carcinoma [8,9,10,11,12,13,14,15,16,17]. Exosomes are vesicles of 30–150 nm diameter, loaded with unique cargo, including proteins, nucleic acids, lipids, and metabolites, that could predict the cells of their origin [7]. Exosomal membranes are enriched with endosome-specific tetraspanins, such as CD9 and CD63 [7,18].

CD9 protein is a member of the transmembrane 4 superfamily [7]. Some reports indicate the tendency of exosome markers in cancer progression. For example, in clinical studies, reduced expression levels of the CD9 are correlated with progression in a range of cancers [7].

Recently, the potential role of exosomal protein value in the diagnosis and risk stratification of PCa by assessment of the level of exosomal proteins in plasma has been demonstrated [19,20].

It has been shown that the CD9 surface marker is less expressed compared to CD63 in serum exosomes from PCa patients [19].

Recently, it was shown that the CD63 concentration isolated from plasma exosomes in patients with PCa was significantly higher compared to that in patients with benign prostate hyperplasia (BPH) [20]. In addition, it was shown that the CD63 level in urine samples was significantly increased in patients with PCa [21].

Previous studies have shown the value of exosomal CD9 and CD63 for the prostate cancer diagnostics in blood and urine. However, CD63 and CD9 expression in the tissue of prostate cancer and benign hyperplasia is still poorly understood [7,20,21,22].

Failure of effective DNA damage repair is a characteristic of cancer. The DNA mismatch repair (MMR) system recognizes and repairs genomic mismatches that occur on DNA during replication [23,24]. However, previous studies have shown that the prevalence of MMR deficiency in all PCa cases is only about 15% [23]. A recent study of MSH2 protein expression in 1133 PCa patients identified loss in 1.1% of patients, significantly detected in grade group 5 with disease progression association [25].

Furthermore, it is of particular importance to evaluate the associations of prostate cancer histopathological features and loss of MMR proteins, since personalized treatment could be beneficial for some patients with PCa.

The aim of the current study was to analyze CD63, CD9, and MMR protein expression in prostate tissue in patients with prostate cancer and benign hyperplasia and its correlation with ISUP grade groups and progression-free survival.

## 2. Materials and Methods

### 2.1. Patients Characteristics

The study was retrospective. Altogether, 92 patients with prostate acinar adenocarcinoma undergoing radical prostatectomy (RP) and 20 patients undergoing fine-needle biopsy with histopathologically confirmed BPH diagnosis (control group) in the year 2013–2015 were enrolled in the study. The study was performed in accordance with the Declaration of Helsinki. The study was approved by the Ethics Committee of Institute of Cardiology and Regenerative Medicine, Riga, Latvia.

Tissue samples have been collected from the Biobank of Riga East Clinical University Hospital & Institute of Clinical and Preventive Medicine, University of Latvia. All patients gave written consent to participate in the scientific research.

All the PCa patients in our study have been followed up till disease progression and/or for a maximum of 5 years of timeframe.

### 2.2. Histopathological Examination

The histopathological evaluation of PCa tissue was performed according to the guidelines of the current World Health Organization (WHO) classification of Tumors of the Urinary System and Male Genital Organs and CAP (College of American Pathologist) PCa protocol. Cancer type, Gleason grading, grade group, and cancer invasiveness were assessed. The cancer TNM staging was performed according to the 8th American Joint Committee on Cancer staging manual. AJCC prognostic stage groups were assessed by incorporating TNM, PSA level, and grade groups.

### 2.3. Tissue Immunohistochemistry

Paraffin-embedded tissue specimens were retrieved from the Biobank of Riga East Clinical University Hospital. Specimens were cut into 3 µm thick sections, and the slides were stained with hematoxylin and eosin to evaluate histopathological changes. Antigen retrieval was achieved by incubating the slides with Tris/EDTA buffer at pH = 9.0 for 30 min in a scientific microwave. The slides were then incubated overnight at 4 °C with mouse monoclonal CD9 (AbCam, ab215), mouse monoclonal CD63 antibody (AbCam, ab215891), rabbit monoclonal antibody MSH2 (AbCam, ab227941, dilution 1:500), rabbit monoclonal antibody MSH6 (AbCam, ab273076), rabbit monoclonal antibody MLH1 (AbCam, ab23844, dilution 1:500), and rabbit monoclonal antibody PMS2 (AbCam, ab110630, dilution 1:100). Antibody binding was detected using the EnVision reagent following the manufacturer’s instructions (DAKO). Immunostained slides of each histology sample were scanned at a magnification of ×20. The whole area scanned of each slide was analyzed with *Image Analysis Quant Center* (3DHistech). CD63, CD9, and MMR protein expression (MSH2, MSH6, MLH1, and PMS2) were evaluated by the intensity of staining and the percentage of stained cancer cells and stromal cells, respectively: the intensity was given scores of 0–3 (0 = no, 1 = weak, 2 = moderate, and 3 = intense), and the percentage of immunopositive cells was given scores of 0–3 (0 = 0%, 1 = 10%, 2 = 20–30%, and 3 = 40–100%). The two scores were multiplied to obtain the decisive result of 0–9. Expressions were considered positive in tumor cells when scores were 2 or more and negative when scores were 0–1. Evaluation was made by two double-blinded independent observers who were unaware of clinical data and outcome.

### 2.4. Statistical Analysis

Values were expressed as the mean (range). The Fisher exact test or the chi-square test was used to evaluate the association between categorized variables. Associations between CD63, CD9, and MMR expression and clinicopathological findings were analyzed using the chi-square test. Progression-free survival (PFS) was defined as the time from operation to the time of disease progression. PFS curves were estimated by the Kaplan–Meier method and compared using the log-rank test. Multivariate analysis was carried out using the cox proportional hazard model. The estimated PSA value decrease within 6 weeks post RP was according to guidelines defined as <0.1 ng/mL. According to guidelines, we used a PSA value greater than 0.4 ng/mL as the threshold post RP that best predicts metastasis and in our study is defined as PSA relapse or biochemical recurrence. The local or distant metastasis would be subsequently detected using imaging diagnostics (for example, bone scan, abdominopelvic CT, or MRI). *p*-Values less than 0.05 were statistically significant. SPSS 21.0 version software was used for the statistical analysis.

## 3. Results

Altogether, 112 patients were enrolled in the study (shown in Table 1), of which 92 patients were with PCa and 20 patients were with BPH. Low-grade PCa (grade groups I and II) was observed in 56 patients, and high-grade PCa (grade groups III–V) was observed in 36 patients. None of the patients had distant metastasis. PSA value > 0.4 ng/mL as the threshold post radical prostatectomy (RP) and primary and/or imaging data subsequently were used as an indicator for tumor progression [1]. Using imaging diagnostics subsequently, the local and/or distant metastasis secondary have been detected within a maximum of 5-year follow-up in 18 patients (19.50%).

### 3.1. CD63 Expression in Prostate Tissue

**Figure 1:**
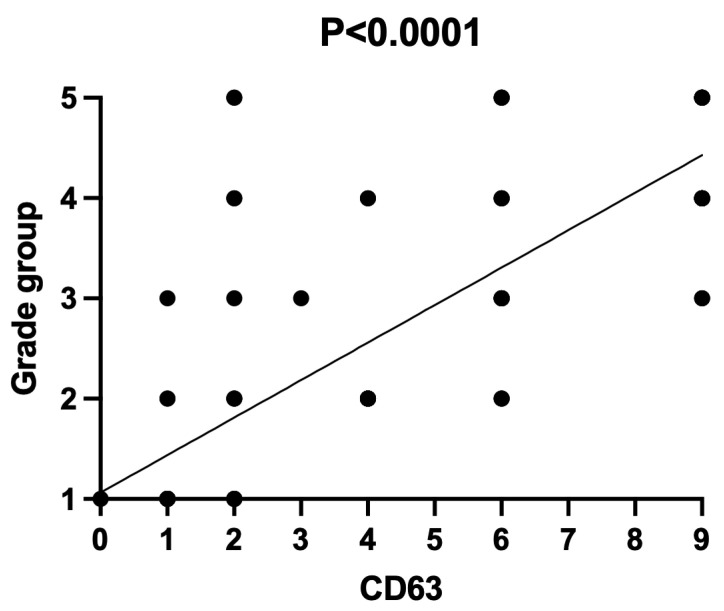
Association between CD63 expression and grade group via Pearson’s chi-squared test (χ^2^). Pearson’s χ^2^ = 0.59; *p* < 0.0001.

**Figure 2:**
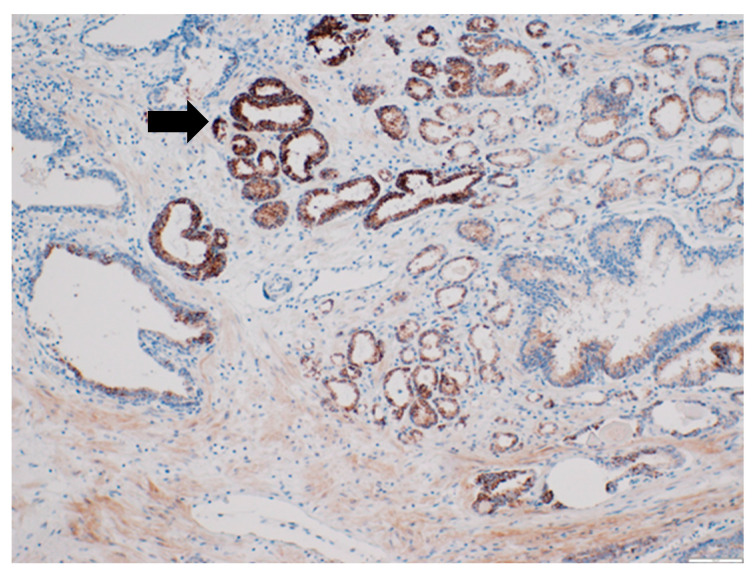
Representative photomicrograph of CD63 expression in high-grade prostate cancer. The black arrow indicates CD63 protein expression. Immunohistochemical staining method; magnification ×200; scale bar 100 µm.

CD63 was mainly expressed on the cell membrane of PCa cells (less in the cytoplasm). There was no significant difference between CD63 expression between BPH and PCa. However, there was a tendency of increased CD63 expression in patients with PCa (4.21 (0–9) vs. 2.55 (0–9) score; *p* = 0.09). However, when the low- and high-grade PCa patients were independently analyzed, the CD63 expression was significantly higher in patients with PCa Grades III–V (shown in Figure 1) compared to those with PCa Grades I–II (respectively, 6.24 (0–9) vs. 1.57 (0–6) score), and there was an association between CD63 expression score and PCa grade groups (Pearson’s χ^2^ = 0.59; *p* < 0.0001), as shown in Figure 1 and Figure 2. The median PFS was significantly longer in patients with low CD63 expression compared to those with high CD63 expression (respectively, 42.50 and 26.50 months; *p* = 0.018).

**Figure 3:**
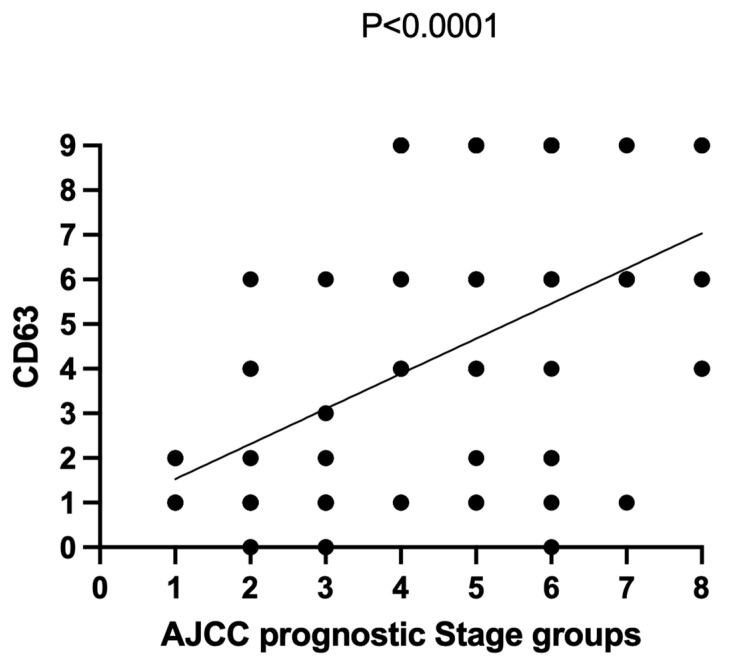
Association between CD63 expression and AJCC prognostic stage groups via Pearson’s chi-squared test (χ^2^). Pearson’s χ^2^ = 0.27; *p* < 0.0001.

An association between CD63 expression and AJCC prognostic stage groups was observed (Pearson’s χ^2^ = 0.27; *p* < 0.0001), as shown in Figure 3.

### 3.2. CD9 Expression in Prostate Tissue

**Figure 4:**
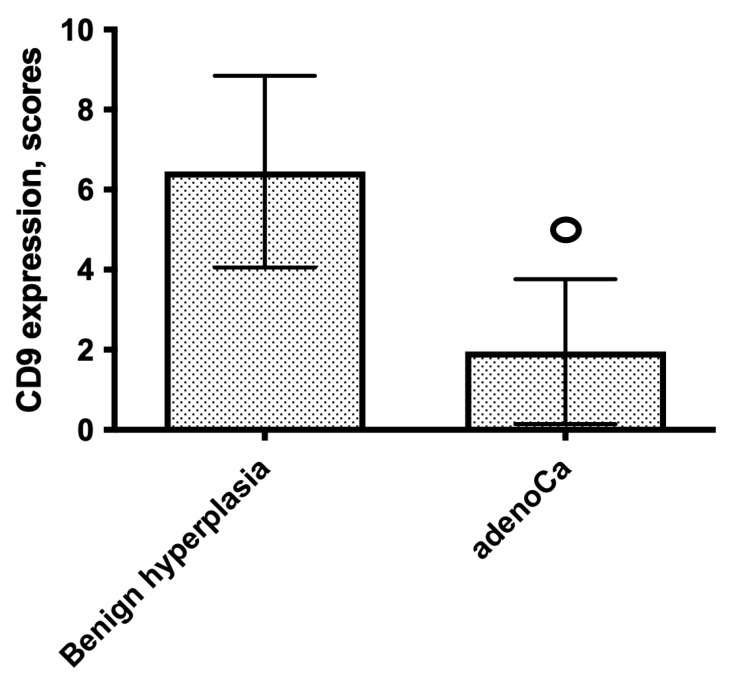
Comparison of CD9 expression in patients with prostate benign hyperplasia and acinar adenocarcinoma; ^ₒ^ *p* < 0.0001; Mann–Whitney U test.

**Figure 5:**
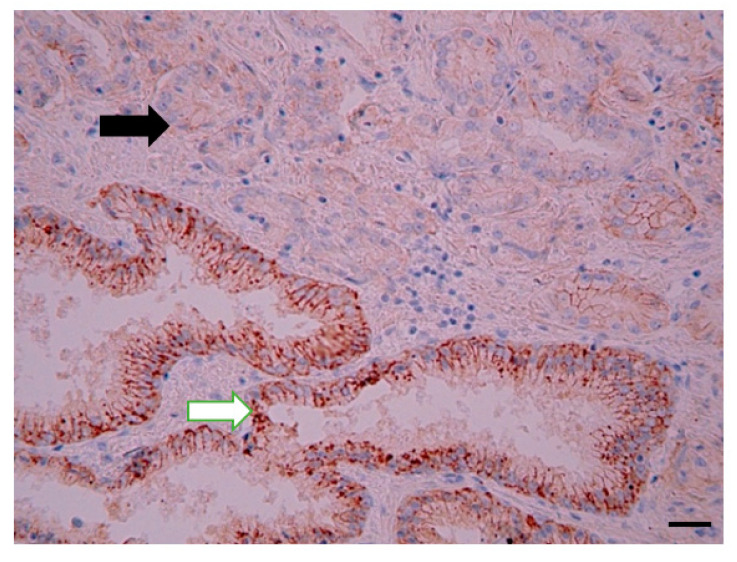
Loss of CD9 expression in prostate acinar adenocarcinoma. The black arrow indicates loss of CD9 protein expression in acinar adenocarcinoma, and the white arrow shows preserved CD9 expression in benign prostate glands. Immunohistochemical staining method; magnification ×400; scale bar 100 µm.

CD9 staining was cytoplasmic, vesicular, predominantly focal, and mainly located apically. In BPH, moderate or intense staining was observed whereas the expression of CD9 in cancer tissue was almost mild or absent. CD9 expression was significantly decreased in PCa compared to that in the control group: expression score 1 (0–9) vs. 6 (2–9); *p* < 0.0001 (shown in Figure 4 and Figure 5). The median PFS in patients with high CD9 expression (score 4–9) was significantly longer compared to that in patients with low CD9 expression (score 0–3) (respectively, 43.00 and 28.50 months; *p* = 0.016). An association between CD9 expression and AJCC prognostic stage groups was not observed (*p* > 0.05).

**Figure 6:**
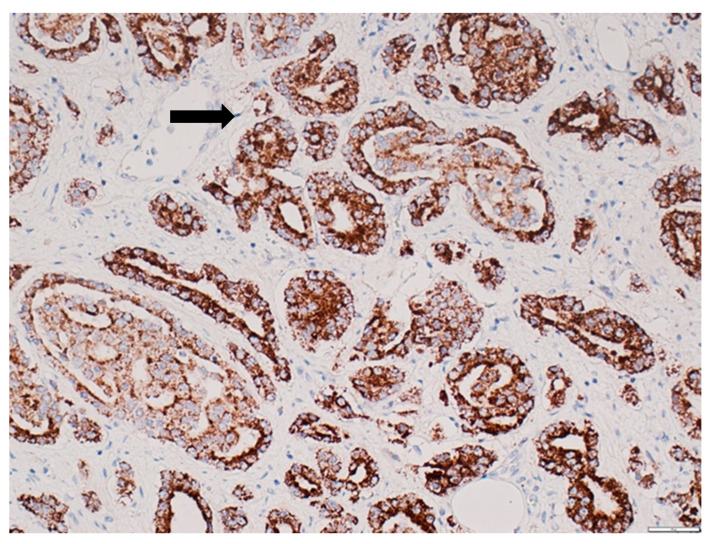
MSH-2 expression in prostate acinar adenocarcinoma. The black arrow indicates immunopositive cells. Immunohistochemical staining method; magnification ×200; scale bar 100 µm.

MMR (MSH2 (shown in Figure 6), MSH6, MLH1, and PMS2) expression in prostate tissue.

MMR expression was absent in 10 patients (10.86%) of 92 PCa patients (2 patients with grade group II, 5 patients with grade group IV, and 3 patients with grade group V).

**Figure 7:**
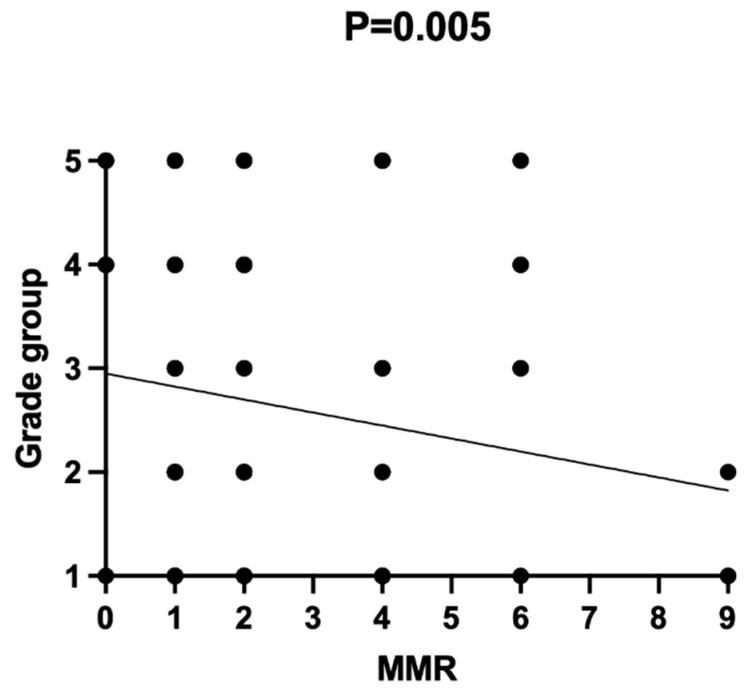
Association between MMR expression and grade group via Pearson’s chi-squared test (χ^2^). Pearson’s χ^2^ = 0.0088; *p* = 0.005.

**Figure 8:**
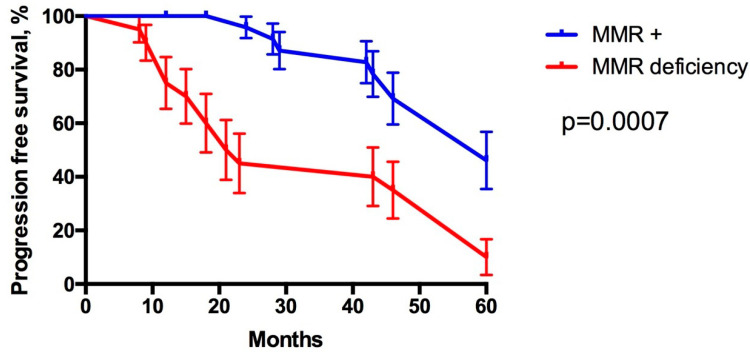
Progression-free survival of prostate cancer patients with MMR deficiency (red line) and maintained MMR expression (blue line). Kaplan–Meier method using the log-rank (Mantel–Cox) test; *p* = 0.0007.

Our study demonstrated the loss of MMR expression in 8/36 (22.22%) of high-grade PCa patients and 2/56 (3.57%) of low-grade PCa patients. MMR was present in all cases of BPH (mild-to-moderate staining). The study revealed an association between the loss of MMR proteins and grade groups (Pearson’s χ^2^ = 0.0088; *p* = 0.005), as shown in Figure 7. The median PFS in patients with MMR deficiency was significantly shorter compared to that in patients with preserved MMR expression (respectively, 22.00 and 60.00 months; *p* = 0.0007), as shown in Figure 8.

An association between MMR expression and AJCC prognostic stage groups was not observed (*p* > 0.05).

## 4. Discussion and Conclusions

PCa is one of the most common malignancies in developed countries [1]. Most PCa cases are latent, which remain as local disease. It is necessary to identify which PCas are destined to progress and would benefit from an early radical treatment [1,2]. PSA currently is the most widely used test to detect PCa. However, its limited specificity has led to scientific research of novel biomarkers and testing methods [1,2,4,5,20,21].

Exosomes are cell-derived vesicles that are present in perhaps all eukaryotic fluids, including blood, urine, and cultured medium of cell cultures [8,26]. In malignancies, the regulatory circuit of exosome homeostasis is damaged and promotes cancer cell survival and metastasis [17,27]. Exosomes act as mediators for intercell communication and are a potential source of biomarkers for many diseases, including PCa [7]. Exosomal proteins might be the potential biomarkers for PCa and could be detected in blood plasma and urine [7,19]. Our study is one of first research investigating the clinico-pathological significance of CD63 and CD9 expression in PCa tissue, since previous studies have focused on the role of CD63 and CD9 expression in plasma and urine samples [20,21,28].

However, these proteins have not been investigated in the tissue of prostate cancer and benign hyperplasia and, therefore, it is still unclear whether the plasma and urine exosomal proteins biomarkers are correlated with tissue models. This is of particular importance since the gold standard for prostate cancer diagnostics is the histopathological examination and, therefore, the assessment of prostate cancer tissue biomarkers could guide the potential personalized diagnostics, treatment, and prognosis.

Our study demonstrated that CD63 but not CD9 expression was significantly higher in patients with PCa Grades III–V (high-grade PCa) compared to those with PCa Grades I–II (low-grade PCa). In addition, CD63 expression correlated with AJCC prognostic stage groups. Furthermore, PFS was significantly longer in patients with low CD63 and high CD9 expression.

Previous studies have shown that patients with lower plasma CD63 concentrations have greater prostate volumes and lower Gleason scores [20,28], which, in relation to prostate-cancer-grading-inclusive Gleason scoring, is consistent with our findings in tissue model.

Our study for the first time demonstrated simultaneous assessment of CD63, CD9, and MMR protein expression in patients with prostate cancer and benign hyperplasia; previous studies have been focused on serum and urine assessment.

Both CD9 and CD63 are exosomal proteins. However, according to our findings, it is intriguing why CD63 is upregulated but CD9 downregulated in PCa.

Bijaya et al. found that the CD9 surface marker is less expressed compared to CD63 in serum exosomes from PCa patients [19]. This may indicate the exosomal sub-population theory, regarding exosome concentration, heterogeneous surface peculiarities, and possibility of multiple phenotype coexistence [19]. In contrast, other studies (for example, by Mizutani et al.) have found exosomes representing a higher amount of CD9 surface marker in advanced and chemo-resistant PCa [29].

This could in part explain the differences in CD9 and CD63 expression in the prostate tissue in our study.

Approximately 10% of advanced and metastatic prostate carcinomas have single-nucleotide mutations, almost always due to underlying inactivation of genes in the MMR system, for example, *MSH2*, *MSH6*, *MLH1*, or *PMS2*, and are often accompanied by microsatellite instability (MSI). MMR and MSI play an important role in the clinical relevance of various malignancies, with the recent Food and Drug Administration (FDA) approval of the PD-1 inhibitor pembrolizumab to treat metastatic cancers of all histologic types with MMR deficiency or MSI. A cost-effective method for routine MMR deficiency assessment could be tissue immunohistochemical investigation [1,30,31].

MSH2 loss has been demonstrated in up to 10% of prostate cancer cases, mostly in high-grade tumors [32].

Our study demonstrated that MMR expression was absent in 10.86% of the patients: 8/36 (22.22%) of the high-grade PCa patients and 2/56 (3.57%) of the low-grade PCa patients.

In addition, the negative correlation of loss of MMR expression and grade groups was revealed in our study.

Only a few studies have reported the incidence of MMR deficiency in prostate cancer ranges from 1.2 to 22.7% [30,31,32]. MSH2 loss was more frequently observed mainly in in high-grade PCa patients [32]. In contrast, other studies have not demonstrated significant associations between MMR loss and grade groups [25,33,34,35]. The role of DNA MMR genes in PCa is still a matter of debate, as genetic alterations leading to MSI are incompletely defined in PCa [30].

Our findings confirmed previous observations by demonstrating that the loss of MMR is observed mainly in high-grade PCa. Furthermore, MMR was present in all cases of benign prostate hyperplasia.

In the study of Gonzalez et al., loss of MMR proteins was observed in 8% of the MSH2 group, 5% of the MLH1 group, and 2% of the PMS2 group, with no statistical differences between the groups [30].

In the study from Fraune et al. MMR deficiency was observed in 3.5% of the cases [31].

In the study by Gonzalez et al., MSH6 overexpression was found in about 42% of prostate tumors, with a cancer aggressiveness association. It could be suggested that MSH6 protein overexpression represents increased DNA damage [30]. However, the same impact on MMR overexpression has not been observed in our study, which could be explained by the fact that only some patients in our group had locally advanced disease.

It has been suggested that MSH6 could be a biomarker of genomic damage and aggressiveness in PCa [30]. Our findings, in part, support this observation by demonstrating that the loss of MMR expression is predominantly observed in high-grade PCa patients.

However, some studies demonstrated that MSH6 expression could be downregulated not only in PCa but also in BPH, leading to the hypothesis about inherited cases [30,33,34,35]. The loss of MSH6 expression in BPH has not been demonstrated in our study.

In conclusion, high-grade PCa was characterized by increased expression of CD63 but downregulation of CD9 protein expression and downregulation of MMR, compared to low-grade cancer, which correlated with progression-free survival. The loss of MMR characterized high-grade PCa. MMR protein expression was present in all cases of BPH.

CD63, CD9, and MMR routine tissue IHC detection might be a prognostic marker for patients with PCa.

## Figures and Tables

**Table 1:** Clinical characteristics of patients.

Age	63.23 (43–85) Years
Grade group I	24 patients
Grade group II	32 patients
Grade group III	18 patients
Grade group IV	10 patients
Grade group V	8 patients
pT2	66 patients
pT3a	18 patients
pT3b	6 patients
pN0	81 patients
pN1	8 patients
Stage group I	6 patients
Stage group IIA	9 patients
Stage group IIB	15 patients
Stage group IIC	14 patients
Stage group IIIA	8 patients
Stage group III B	24 patients
Stage group IIIC	8 patients
Stage group IVA	8 patients

## Data Availability

All authors in sending the manuscript together with illustrations and tables agree to the automatic and free transfer of copyright to the publisher, allowing for the publication and distribution of the material submitted in all available forms and fields of exploitation, without limits of territory or language, provided that the material is accepted for publication. At the same time, the authors accept that the submitted work will not be published elsewhere and in any language without the earlier written permission of the copyright holder, i.e., the publisher and the editor-in-chief. All authors provide a data availability statement in this article.
